# Diagnostic Dilemma in Two Cases of Hyperandrogenism

**DOI:** 10.1155/2018/9041018

**Published:** 2018-06-27

**Authors:** Ibrahim Alali, Lilianne Haj Hassan, Ghadeer Mardini, Nermeen Hijazi, Lama Hadid, Younes Kabalan

**Affiliations:** ^1^Endocrinology Department, Al-Assad University Hospital, Damascus University, Syria; ^2^Endocrinology Department, Al-Mouwassat University Hospital, Damascus University, Syria

## Abstract

Hirsutism is a common endocrine complaint affecting about 10 percent of women. It may be caused by multiple etiologies including adrenal and ovarian disorders. Usually, it is a result of a benign entity such as PCOs and idiopathic hirsutism. However, sometimes especially when it is severe and rapid in progression an androgen-secreting tumor should be excluded. Sertoli-Leydig cell tumors constitute fewer than 0.5 percent of ovarian tumors and it may be benign or malignant. In this article, we present two cases of hyperandrogenism caused by occult ovarian Leydig cell tumors. one of them was confounded by the presence of coincidental bilateral adrenal nodules that complicated the diagnostic process. Tumor dissection was curative in both cases and the diagnosis was confirmed by pathological and hormonal testing after surgery.

## 1. Introduction

Hirsutism is known as excess male-pattern hair growth in women; it produces a significant emotional stress regardless of its etiology [1].

Endogenous androgens are made by the adrenal glands and the ovaries in both post- and premenopausal women [2].

Hirsutism is usually caused by benign etiologies such as polycystic ovary syndrome PCOs and idiopathic hirsutism. However, when it is severe and/or associated with virilization, an androgen-secreting tumor should be excluded [3].

These tumors may originate from adrenals or ovaries, where they arise from sex cord such as Sertoli-Leydig cell tumors [4].

They may be so small in contrast to adrenal tumors which are often large in size and aggressive in behavior.

Due to the high incidence of adrenal incidentalomas, the incidence of an adrenal mass does not exclude the ovarian origin of androgen secretion and then the differential diagnosis is sometimes so complex [5].

Here we describe two cases of hyperandrogenism in post- and premenopausal women caused by androgen-secreting ovarian tumors.

## 2. Case 1 Presentation

A 60-year-old woman came to the endocrinology clinic with a complaint of rapidly progressive signs and symptoms of hyperandrogenism over 6 months. She mentioned hirsutism noticed especially in the face and chin, hair loss that took a male-pattern baldness in all over the head, deepening voice, and increased libido.

She had no galactorrhea, muscle weakness, hyperpigmentation, bruising, weight loss, or anorexia.

She was married, housewife, and smoker (5 pack-years), got 6 children, did not consume alcohol; she had regular menses since puberty until she had amenorrhea 22 years ago after hysterectomy (because of leiomyoma). She was diagnosed with hypothyroidism 15 years ago treated with L-Thyroxine (700 *μ*g∖weekly) and osteoporosis 7 years ago treated with Calcium supplements + alendronate 70 mg weekly. She denied the use of any drugs that may cause hyperandrogenism.

**Figure 1:**
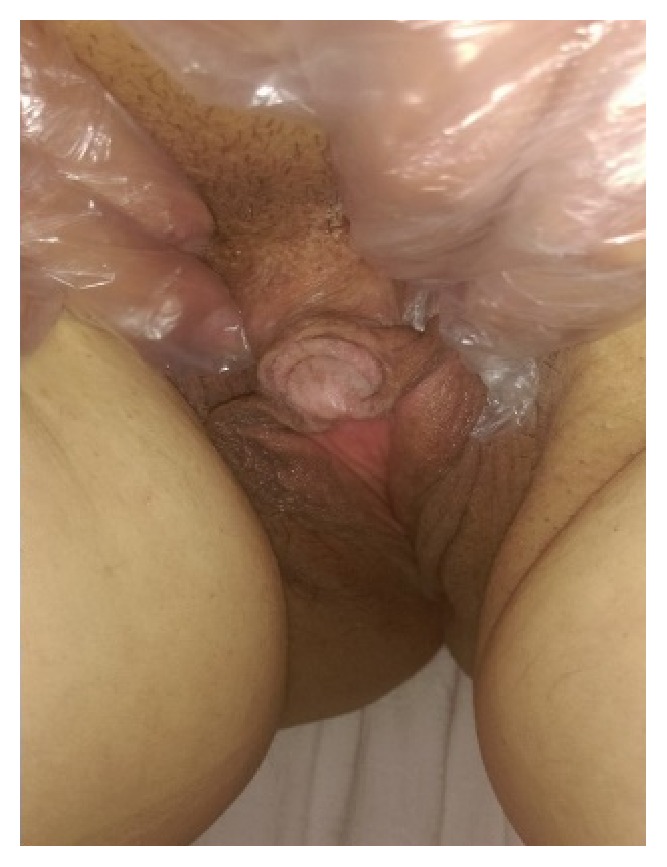
Clitoromegaly.

On examination, the patient seemed well. The blood pressure was 120/80 mm Hg, the pulse 83 beats per minute, the height 154 cm, the weight 72 kg, and the body mass index (BMI; the weight in kilograms divided by the square of the height in meters) 30.2 (obesity class I). The Ferriman-Gallwey score for hirsutism estimation was 6 (4 in the chin, 2 in upper lip); she had acanthosis nigricans, frontal baldness, and clitoromegaly (2 cm by 3 cm) as shown in (Figure 1). Except for a cesarean scar in the abdomen, the rest of examination was unremarkable.

Laboratory studies revealed a hemoglobin concentration of 15 g/dL, serum sodium level of 141 mEq/L, and potassium level of 4.5 mEq/L. An automated chemistry panel showed normal findings. Hormonal studies were as in Table 1.

**Figure 2:**
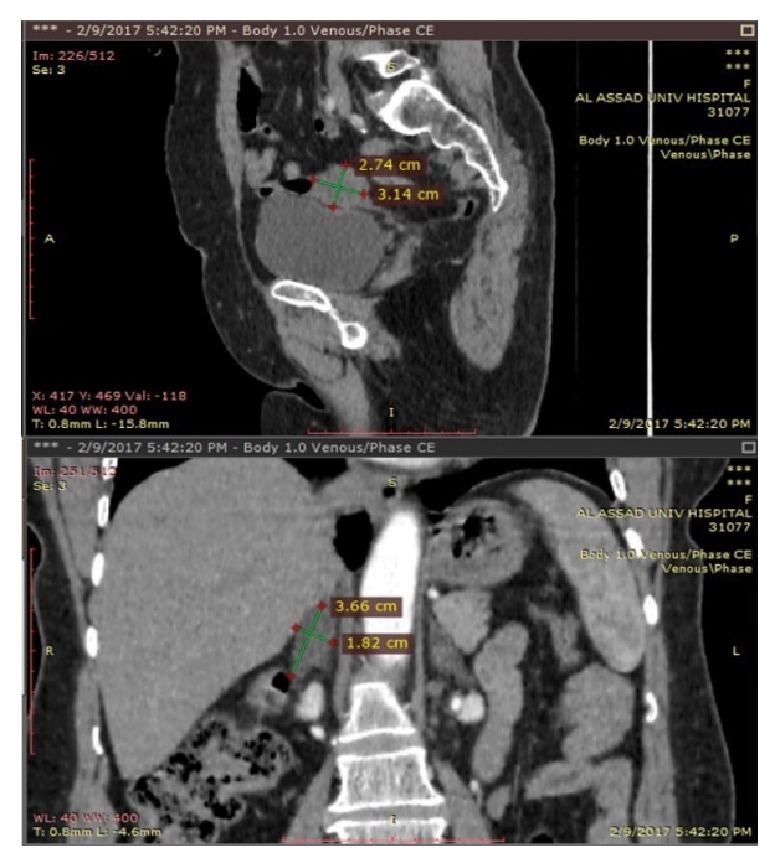
On the top, the figure shows sagittal CT section of the left ovary measures 2.74 cm by 3.14 cm. On the bottom, it shows coronal section adrenals with the largest nodule in right adrenal gland measures 3.66 cm by 1.82 cm with a left adrenal nodule.

Transvaginal ultrasonography showed that uterine and left ovary was removed, right ovary measured 2.1 cm by 2 cm by 4.5 cm with a volume of 7.8 cm^3^. Abdominal computed tomography (CT) showed bilateral adrenal nodular hyperplasia as in (Figure 2). All adrenal function tests (hypo- and hypersecretion) were proved to be normal: 24-hour urine normetanephrine 47.3 *μ*/24hours (up to 600), metanephrine 107 *μ*/24hours (up to 350), 8 A.M cortisol 13.02 *μ*g/dL, adrenocorticotrophic hormone ACTH 19.69 pg/mL (7-63), and 11 P.M cortisol 1.66 *μ*g/dL

In order to distinguish ACTH-dependent hyperandrogenism from other causes of hyperandrogenism, a 48-h low-dose (2mg) dexamethasone-suppression test was carried out [6], without a decrease in testosterone value (10.94 ng/mL) though enough cortisol suppression at the end of the test 0.58 *μ*g/dL.

**Figure 3:**
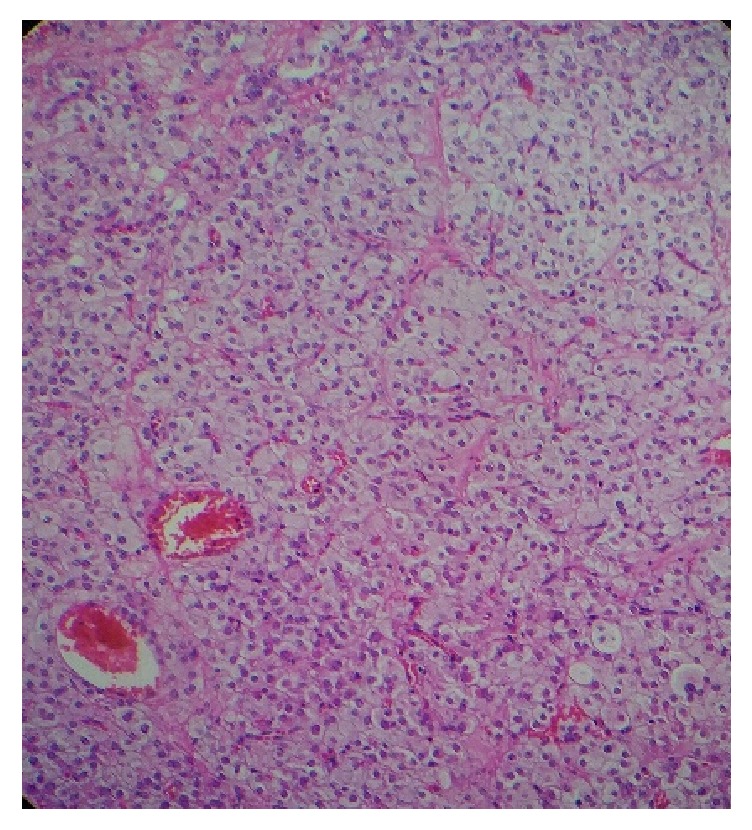
Leydig cell tumor composed by granular cells with eosinophilic cytoplasm and round nuclei (hematoxylin and eosin stain).

In such cases the catheterization of the adrenal and ovarian veins may be useful in identifying the source of hyperandrogenism but it was not available at our center. Since the lack of dexamethasone-induced inhibition of testosterone was suggestive of an ACTH-independent etiology mainly ovarian, and the patient was postmenopausal, the decision of laparoscopic oophorectomy was made. The pathology report confirmed the diagnosis of 2.8 cm Leydig cell tumor (Figure 3). Testosterone was performed 72-hour postsurgery and it was 0.03 ng/mL. 17 hydroxy-progesterone and testosterone were performed 1 month later and they were in normal limits.

## 3. Case 2 Presentation

A 39-year-old woman came to endocrinology clinic with a complaint of hirsutism started 4 years ago, alongside with oligomenorrhea followed by amenorrhea two years ago. There was no temporal baldness or deepening voice.

The patient was treated for a period of 3 months with combined oral contraceptive pills COCP and cyproterone acetate without improvement in symptoms, 6 months earlier to admission.

She was married, got 3 children and was nonsmoking or alcohol consuming. She was diagnosed 5 years ago with rheumatoid arthritis and treated for only one month with prednisolone and methotrexate.

On examination, she seemed well. The blood pressure was 120/80 mm Hg, the height 155 cm, the weight 65 kg, and the body mass index BMI 27.1 (overweight). The Ferriman-Gallwey score for hirsutism estimation was 16 (4 points for each chin, upper lip, low abdomen, and medial thigh), clitoromegaly (1 cm by 0.5 cm); she had no acanthosis nigricans or frontal baldness. Otherwise, she had normal findings.

Laboratory studies revealed a hemoglobin concentration of 10.3 g/dL, ferritin 10 ng/mL, serum sodium level of 138 mEq/L, and potassium level of 4.15 mEq/L. An automated chemistry panel showed normal findings except for fasting glucose 119 mg/dL. She started metformin therapy and ferrous replacement. Hormonal studies were as in Table 1.

**Figure 4:**
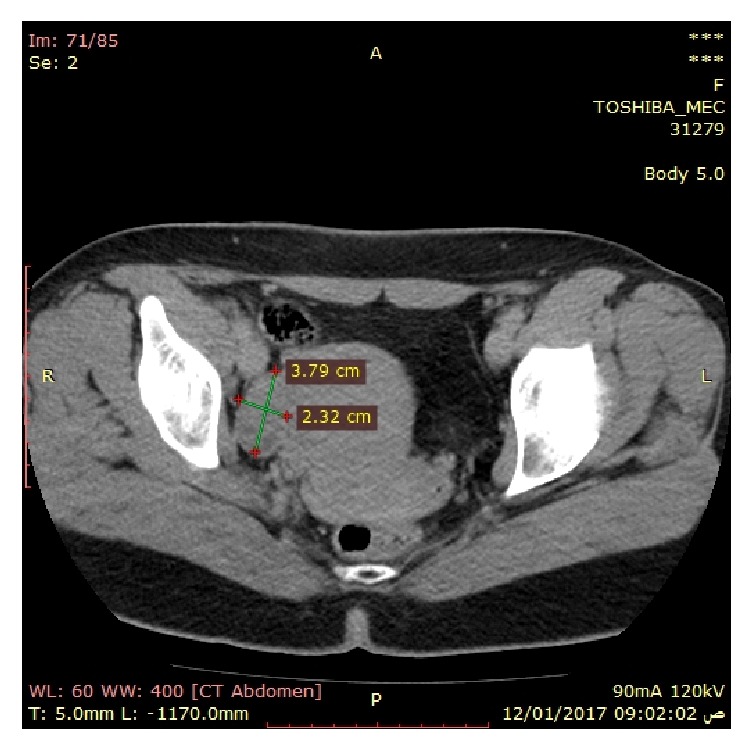
Right ovary measures 3.8 by 2.3 cm.

Transvaginal ultrasonography showed that ovaries measured 3.3 by 2 cm and 3.2 by 2 cm for right and left ovary, respectively, without masses. CT scan for adrenals was within normal also and right ovary measured 3.8 by 2.3cm as shown in Figure 4.

Since catheterization of the adrenal and ovarian veins was not available, the diagnostic and therapeutic options were explained to the patient and giving that she was not interested in future fertility, she underwent laparoscopic exploration for oophorectomy.

**Figure 5:**
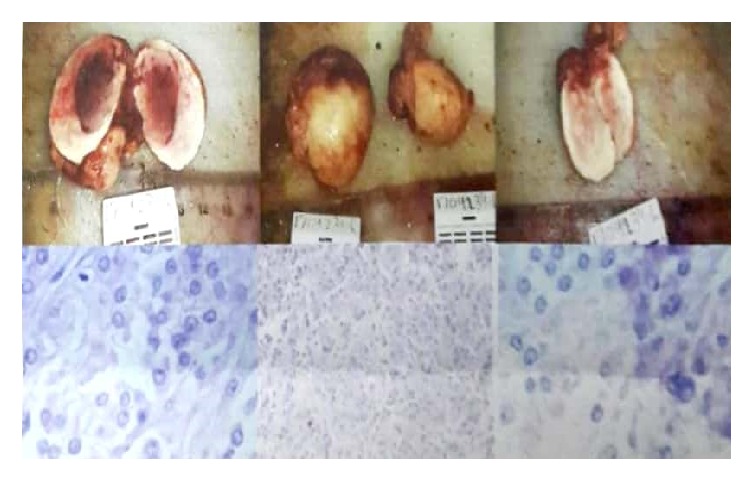
Upper left shows Leydig cell tumor of the right ovary. Upper right shows normal left ovary; microscopic examination shows cells abundant eosinophilic cytoplasm. Reinke crystals are noted.

Pathologic report sowed 2.5 cm of Leydig cell tumor in the right ovary, while the left ovary was within normal as shown in (Figure 5). Testosterone was normalized after surgery.

## 4. Discussion

Androgen-secreting tumors typically come with quickly progressive hyperandrogenism resulting in virilization. These tumors may ascend from adrenal glands or ovaries representing the least common cause of the hyperandrogenism in women with a prevalence of 0.2% [7].

Androgen-secreting adrenal tumors are very infrequent, large, and aggressive and usually associated with high cortisol levels, while purely androgen-secreting adrenal tumors are very rare [8, 9].

Virilizing ovarian tumors are rare medical condition representing less than 0.2% of all causes of hyperandrogenism and fewer than 1% of all ovarian tumors [10].

Although DHEAs level might help to differentiate ovarian from the adrenal source of hyperandrogenism, adrenocortical tumors might sometimes present with normal DHEAs level [11]. Furthermore, ovarian tumors have been rarely reported to be associated with a high DHEAs level which makes final diagnosis very challenging [12].

Leydig cell tumors are a very uncommon type of virilizing ovarian tumors (less than 0.1 % of ovarian tumors) and might occasionally be small enough to pass detection even with careful radiological studies [10] leading to bilateral oophorectomy as both a diagnostic and therapeutic approach [13].

Here we represented two cases of occult Leydig ovarian tumors. One of these cases was associated with bilateral nodular adrenal hyperplasia; these cases highlight the challenges in the diagnosis of Leydig cell tumors.

In the first case, the patient denied the use of any drugs that may lead to hyperandrogenism and because of her severe symptoms, other etiologies should be excluded [14].

In women with PCOS symptoms generally begin in puberty and gradually develop through reproductive years [14] so new onset virilization symptoms and very high testosterone levels proposed to rule out PCOS. Patient's age and absence of adrenal insufficiency excluded classical congenital adrenal hyperplasia CAH, but not the nonclassic form of CAH, although it was theoretically excluded because of the recent onset and severity of symptoms, very high values of testosterone, and the absence of testosterone suppression after dexamethasone. However, we depended on 17 OH progesterone monitoring after surgery to make a definite diagnosis.

A suppression test with dexamethasone was performed [6], and plasma cortisol levels decreased, but total testosterone inhibition did not occur discarding the functional adrenal hyperandrogenism and indicated tumorous origin, with the absence of cortisol cosecretion and normal DHEAS values suggested the presence of an occult ovarian tumor.

Catheterization of both the adrenal and ovarian veins was not suggested because it is unavailable at Al-Assad Center so the patient went to oophorectomy and the diagnosis was made.

The 17OH progesterone returned to normal one month after surgery confirming the ovarian source and excluding nonclassic CAH.

Some cases of unilateral adrenal nodule correlation with Leydig cell tumor were reported [5, 15], but according to our knowledge, this is the first reported case of coincidence bilateral adrenal incidentalomas with Leydig cell tumor in a postmenopausal woman.

In the second case, the absence of adrenal or ovarian mass suggested the use of catheterization of both the adrenal and ovarian veins, but this procedure was not available at Al-Mouwassat Center and the patient was not interested in another pregnancy so after discussing the possibility of an occult ovarian tumor as a cause of her complaint she decided to have bilateral oophorectomy to make a final diagnosis.

The establishment of the precise cause of androgen excess may not always be apparent, necessitating engagement of a combination of clinical talents complemented with appropriate laboratory and imaging techniques [16].

## Figures and Tables

**Table 1:** Hormonal study at initial assessment and normalization of androgens one-month postsurgery.

Test (Normal range) unit	Initial evaluation		1 month after surgery	
	Case 1	Case 2	Case 1	Case 2
Testosterone (0.23-0.73) ng/mL	7.05	15	0.03	0.2

DHEAS(60-338)*μ*g/dL	113.4	62.9		

17OHP(0.2-0.9) ng/mL	3.83	-	0.197	-

TSH(0.5-4.5) mIU/L	0.99	1.96		

PRL(up to 25) ng/mL	13.34	16		
